# Molecular Characterization of Differentiated-Resistance MSC Subclones by Single-Cell Transcriptomes

**DOI:** 10.3389/fcell.2022.699144

**Published:** 2022-03-09

**Authors:** Andres Stucky, Li Gao, Shengwen Calvin Li, Lingli Tu, Jun Luo, Xi Huang, Xuelian Chen, Xiaoqing Li, Tiffany H. Park, Jin Cai, Mustafa H. Kabeer, Ashley S. Plant, Lan Sun, Xi Zhang, Jiang F. Zhong

**Affiliations:** ^1^ Department of Medicine, Keck School of Medicine, University of Southern California, Los Angeles, California, CA, United States; ^2^ Medical Center of Hematology, Xinqiao Hospital, Army Medical University, Chongqing, China; ^3^ Neuro-oncology and Stem Cell Research Laboratory, CHOC Children’s Research Institute, Center for Neuroscience Research, Children’s Hospital of Orange County (CHOC), Orange, CA, United States; ^4^ Department of Neurology, Irvine School of Medicine, University of California, Irvine, CA, United States; ^5^ Department of Oncology, Bishan, The People’s Hospital of Bishan District, Bishan, Chongqing, China; ^6^ Stomatological Hospital of Chongqing Medical University, Chongqing, China; ^7^ Department of Hematology, The Second Affiliated Hospital of Chongqing Medical University, Chongqing, China; ^8^ School of Dental Medicine, University of Pennsylvania, Philadelphia, PA, United States; ^9^ Department of Oral and Maxillofacial Surgery, Zhuhai People’s Hospital, Zhuhai Hospital Affiliated with Jinan University, Zhuhai, China; ^10^ Pediatric Surgery, CHOC Children’s Hospital, Department of Surgery, Irvine School of Medicine, University of California, Irvine, CA, United States; ^11^ Division of Pediatric Oncology, Children’s Hospital of Orange County, Orange, CA, United States

**Keywords:** mesenchymal stem cells, cadherin, Yap1, Cdh6, oct4, subclonal tumorigenicity

## Abstract

**Background:** The mechanism of tumorigenicity potentially evolved in mesenchymal stem cells (MSCs) remains elusive, resulting in inconsistent clinical application efficacy. We hypothesized that subclones in MSCs contribute to their tumorgenicity, and we approached MSC-subclones at the single-cell level.

**Methods:** MSCs were cultured in an osteogenic differentiation medium and harvested on days 12, 19, and 25 for cell differentiation analysis using Alizarin Red and followed with the single-cell transcriptome.

**Results:** Single-cell RNA-seq analysis reveals a discrete cluster of MSCs during osteogenesis, including differentiation-resistant MSCs (DR-MSCs), differentiated osteoblasts (DO), and precursor osteoblasts (PO). The DR-MSCs population resembled cancer initiation cells and were subjected to further analysis of the yes associated protein 1 (YAP1) network. Verteporfin was also used for YAP1 inhibition in cancer cell lines to confirm the role of YAP1 in MSC--involved tumorigenicity. Clinical data from various cancer types were analyzed to reveal relationships among YAP1, OCT4, and CDH6 in MSC--involved tumorigenicity. The expression of cadherin 6 (CDH6), octamer-binding transcription factor 4 (OCT4), and YAP1 expression was significantly upregulated in DR-MSCs compared to PO and DO. YAP1 inhibition by Verteporfin accelerated the differentiation of MSCs and suppressed the expression of YAP1, CDH6, and OCT4. A survey of 56 clinical cohorts revealed a high degree of co-expression among CDH6, YAP1, and OCT4 in various solid tumors. YAP1 inhibition also down-regulated HeLa cell viability and gradually inhibited YAP1 nuclear localization while reducing the transcription of CDH6 and OCT4.

**Conclusions:** We used single-cell sequencing to analyze undifferentiated MSCs and to discover a carcinogenic pathway in single-cell MSCs of differentiated resistance subclones.

## Introduction

Mesenchymal stem cells (MSCs) are a vital component of the bone marrow that show the capacity to self-renew and differentiate in culture into various tissues types of mesenchymal origin, such as adipocytes, chondrocytes, osteoblasts, myoblasts, and hematopoietic cells [ (Caplan, 1991) (Pittenger et al., 1999). Although MSCs present numerous therapeutic use opportunities, their potential carcinogenicity remains a vast obstacle hindering the adoption of MSC-based cancer therapies. Donors MSCs support stem cell phenotype derived from acute myeloid leukemia (AML) in a long-term *in vitro* culture system. MSCs protect acute promyelocytic leukemia (APL) cells from apoptosis induced by doxorubicin or serum starvation (Tabe et al., 2004). MSCs induce gastric cancer cell epithelial to mesenchymal transition (EMT), stimulate trans-endothelial and transwell migration *in vitro*, and increase tumor size and liver metastasis *in vivo*. MSCs also promote EMT of breast cancer cell lines (MDA-MB-231, T47D, and SK-Br-3) (Martin et al., 2010). However, the specific mechanisms of MSCs’ cancer-promoting characteristics are still unclear (Sipp et al., 2018).

Here, we hypothesized that MSC-differentiation-resistant subclones become carcinogenic. Such MSCs’ tumorigenicity, commonly in hematologic malignancies (Lee et al., 2019), is similar to leukemia that is derived from hematopoietic stem cell (HSC)-differentiation-resistant subclones [ (Chopra and Bohlander, 2019) [ (Miraki-Moud et al., 2013) (Nowak et al., 2009), thereby eliminating such subclones for enhancement of efficacy (Lee and Li, 2020). We started with the isolation of MSC subclones during osteogenic differentiation, and we found a subpopulation of osteogenesis-resistant MSC subclones *via* single-cell transcriptome analysis (Li et al., 2013). Those differentiation-resistant MSCs (DR-MSCs) subclones resembled leukemia cells, which also are blocked at various stages during differentiation [ (Chopra and Bohlander, 2019) (Li et al., 2013).

We found that the DR-MSCs up-regulated YAP1 gene networks. YAP1, a protein encoded by a gene on human chromosome 11q22, is conserved from *Drosophila* to mammals. It is a downstream effector of the Hippo signaling pathway, essential organ development. In the nucleus, YAP1 upregulates genes involved in stemness, cell proliferation, anti-apoptosis, and EMT [ (Johnson and Halder, 2014) [ (Pan, 2010) (Staley and Irvine, 2012), making it a player in the initiation of cancer and growth of most solid tumors. Generally, YAP1 and its homolog TAZ are suppressed by phosphorylation and cytoplasmic translocation. Otherwise, transcriptional activators can activate them, causing unlimited cell proliferation and tumorigenesis [ (Iglesias-Bartolome et al., 2015) [ (Lee et al., 2013) (Liu et al., 2017). By inhibiting YAP1 *in vitro* and performing a meta-analysis of cancer patient cohorts, we confirmed the role of YAP1 in the tumorigenesis of MSCs. This study sheds light on the role of YAP1 gene networks in MSC tumorigenesis and suggests that the YAP1 network blocks the differentiation of MSC and contributes to the carcinogenesis of MSCs.

## Materials and Methods

### Cell Lines and Groups

Normal human bone marrow-derived mesenchymal stem cells were purchased from ATCC (the Catologue# ATTC PSC-500-012, QC with normal karyotypes, refer to product specifications for details) (March 2018) and cultured according to the vendor’s specifications. To confirm the identity, the cells were genotyped using a PCR-based assay for positive mesenchymal stem cell markers CD10, CD13, and CD29. Cells were tested to ensure the lack of *mycoplasma* contamination. Cells were expanded two passages from stocks. During the experiments, the morphology of all cell lines was routinely checked under a phase-contrast microscope. Cells were thawed and grown for one passage from stocks within 1 month of the initial thaw. During the experiments, the morphology of all cell lines was routinely checked under a phase-contrast microscope. All newly revived cells were seeded in triplicate onto 6-well Eppendorf cell culture plates at ∼ 5,000 cells/cm^2^ and expanded in low-glucose DMEM (Corning) supplemented with 1% penicillin streptavidin (GIBCO). Negativity for *mycoplasma* contamination was determined with Hoechst 33,258 staining under a high-magnification fluorescent microscope. When cells reached 100% confluence, low-glucose DMEM was replaced with the StemPro™ Osteogenesis Differentiation medium (Thermo Fisher Scientific, Canoga Park, CA). Gene expression profiling of MSCs was performed during *in vitro* differentiation. Alongside a control group of unmanipulated MSCs cultured in low-glucose DMEM, we cultured differentiating MSCs in the osteogenic differentiation medium. We profiled all groups at 12, 19, and 25 days post-differentiation. Differentiating MSCs were divided into three subpopulations: 1) differentiated osteoblasts (DO), 2) differentiation/osteogenesis-resistant MSCs (DR-MSCs), and 3) precursor osteoblasts (PO), identified by single-cell capture as described below.

### Alizarin Red Staining

Alizarin Red (2 g) was dissolved in 100 ml of distilled water and mixed, and pH was adjusted to 4.1–4.3 with 0.1% NH_4_OH. The solution was filtered and stored in the dark. Cells were harvested at 12, 19, and 25 days post-differentiation. The medium was carefully aspirated, and cells were washed twice with Dulbecco’s PBS w/o Ca^++^/Mg^++^. PBS was carefully aspirated, and a buffer containing 10% formalin was added to the cell monolayer. After incubation in formalin for 1 h, cells were gently washed with distilled water. Water was aspirated, and enough Alizarin Red to cover the cellular monolayer was added. Cells were incubated at room temperature in the dark for 45 min, after which Alizarin Red was gently aspirated, and cells were washed four times with 1 ml of distilled water. After washing, enough PBS to cover the monolayer was added. The staining intensity was quantified using ImageJ (NIH). Images were converted to grayscale, the background was subtracted, optical intensities were measured, and percentage differentiation was estimated by the ratio of the stained area on the plate to the total area on the plate.

### Single-Cell Capture

Differentiated and non-differentiated cells were incubated with 100 μl of trypsin for 5 min at 37°C, the reaction was terminated by adding 0.5 ml of culture medium, detached cells were collected in 15 ml falcon tubes and centrifuged at 1,000 g to pellet the cells, the supernatant was removed, and cells were washed once with PBS. Following washing, cells were resuspended in 1 ml of PBS and loaded onto a pressure-gated microfluidic single-cell capturing chip. The presence of a single cell in each microfluidic chamber was visually confirmed under a Nikon Eclipse TE300 inverted microscope at 4X amplification. A total of 8 cells were harvested at each timepoint and, after assessing the RNA quality from each cell, a total of 5 cells from each condition were selected for downstream analysis.

### RNA Extraction and Library Preparation

Messenger RNA from whole-cell lysates was isolated using TRIzol^®^ reagent (Life Technologies), and libraries were prepared using an Illumina TruSeq Stranded mRNA library prep kit. Individual MSCs were isolated using a pressure-gated microfluidic chip. Single MSCs were processed using the REPLI-g WTA single-cell (Qiagen). The amplified double-stranded cDNA was fragmented using NEB double-stranded DNA fragments. An Agilent screen tape system was used to quantify 100 ng of fragmented DNA for library prep input. A NEBNext Ultra II DNA library prep kit for Illumina Barcoded libraries was used to process 100 ng of fragmented cDNA, and the resulting products were submitted for RNA sequencing by the Loma Linda University Center for Genomics.

### Single-Cell RNA-Seq and Transcriptome Analysis

Libraries were sequenced on the Illumina HiSeq 4,000 platform (Illumina). The raw reads were filtered by sequencing quality, adaptor contamination, and duplicated reads. Thus, only high-quality reads remained and were used in the genome assembly. An average of 2 million reads was generated for every single cell and 5 million for bulk samples. The RNA-seq data were analyzed with Partek Flow version 4 (Partek Inc.). Bases with Phred scores <20 were trimmed from both ends of the raw sequencing reads and trimmed reads shorter than 25 nt were excluded from downstream analyses. Both pre-and post-alignment quality assessment and quality control were carried out with default settings in the Partek Flow workflow. Trimmed reads were mapped onto human genome hg38 using Tophat 2.0.8 as implemented in Partek Flow with default settings, using Gencode 20 annotation as guidance (gencodegenes.org). Read counts per gene for single-cell samples were normalized using total counts multiplied by 10,000 and transformed using Log e. Bulk samples were normalized using transcripts per million (TPM).

### Differential Gene Expression Analysis

Analyses of transcriptomes from single cells cultured for 25 days were used for the study. Principal component analysis of gene expression of all single cells was performed using the Partek package; identified clusters were then selected for analysis of differential expression using Partek’s Gene Specific Analysis method (genes with <10 reads in any sample were excluded). To generate a list of significantly differentially expressed genes among all samples, the cutoff for significance was defined as the false discovery rate (FDR) adjusted *p*-value (q-value<0.05) and >2-fold change. Gene-specific pathway analysis was performed using Ingenuity Pathway Analysis (IPA) software (Qiagen Bioinformatics). Genes enriched in the most significant pathway (lowest *p*-values) were selected for the evaluation of clinical data meta-analysis.

### Immunofluorescence

HeLa cells were purchased from ATCC (July 2019) and cultured according to the manufacturer’s instructions and specifications. Cells were seeded onto coverslips and cultured with high-glucose DMEM to confluence in 12-well plates. After removing the culture medium, cells were washed three times in PBST (0.1% tween-20) and fixed for 30 min in 4% formalin. The formalin was then removed, and the cells were washed three times in PBST. Unspecific binding was blocked using PBST containing 10% FBS. Cells were then incubated with a 1:500 concentration of rabbit anti-YAP1 antibody (Invitrogen) overnight at 4°C. The following day, cells were brought back to room temperature. The primary antibody was removed. Cells were washed three times with PBST and incubated with secondary goat anti-rabbit conjugated Alexa-fluor 594 for half an hour. Cells were washed and incubated with NucBlue Live-cell stain (Life Technologies). The stain was washed off, and fluorescence was visualized using a Nikon Eclipse Ti2 inverted microscope.

### YAP1 Inhibition by Verteporfin

MSCs were seeded onto coverslips and cultured in 12-well plates. Verteporfin at 2 or 5 μM concentration was administered on Day 1 and Day 4 following stepwise differentiation. Cells were then harvested on Day 12. After that, the percentage of differentiated cells was estimated using Alizarin Red staining, as described above. Expression of YAP1, OCT4, and CDH6 was measured using RT-PCR and normalized to β-actin.

### Tumor Cell Viability After YAP1 Inhibition

Cancer cell viability was assessed using the trypan blue dye exclusion test, and viable cells were then counted using a hemocytometer. HeLa cells were cultured in high-glucose DMEM, passaged, and seeded onto 12-well cell culture plates. Once cells reached confluence, 2 or 5 μM concentration of the YAP1 inhibitor verteporfin was added to the wells for 24 h. Following incubation with Verteporfin, cell viability was assessed as described above. Significance was determined using Student’s t-test.

### Transcription Analysis by qRT-PCR

MSCs and solid tumor cell lines were washed three times with PBST (0.1% tween-20). 1 ml of TRIzol was added to wells, and RNA was extracted according to the manufacturer’s instructions. RNA was reverse transcribed, and levels of YAP1, OCT4, and CDH6 were quantified using primers listed below. qRT-PCR was performed on both MSCs, and solid tumor cell lines using Fast SYBR Green master mix (Thermo-Fisher) in a Bio-Rad CFX Connect Real-time System.

Primers used were: *YAP1* (TAG​CCC​TGC​GTA​GCC​AGT​TA, TCA​TGC​TTA​GTC​CAC​TGT​CTG​T); *OCT4* (CTG​GGT​TGA​TCC​TCG​GAC​CT, CCA​TCG​GAG​TTG​CTC​TCC​A);

*CDH6* (AGA​ACT​TAC​CGC​TAC​TTC​TTG​C, TGC​CCA​CAT​ACT​GAT​AAT​CGG​A).

### Western Blot Analysis

To prepare samples for Western blotting, the cell pellet was washed with 1x PBS (Hyclone) and centrifuged. The supernatant was discarded and the cell pellet then lysed with TBS-T (20 mM Tris pH 7.6, 150 mM NaCl, 1% Triton X-100, with phosphatase inhibitors (10 μg/ml Aprotinin, 10 μg/ml Leupeptin, 5 μg/ml Pepstatin, 1 mM PMSF, 1 mM NaF, 1 mM Na3VO4) added (Li et al., 1996). Alternatively, the cell pellets were lysed in CellLytic™ M–Cell lysis reagent for Mammalian cell lysis and protein solubulization (Sigma-Aldrich, St. Louis, MO, USA) with added cOmplet Tablets (mini EDTA-free Protease inhibitor cocktail EASY pack) (Ref 04 693 159 001, Lot 39968000) (11836170001 Roche, Sigma-Aldrich, St. Louis, MO, USA). A 10% SDS-PAGE gel was prepared for Western blotting. An equal amount of protein taken from the cell lysates was loaded into the gel wells. Samples were then heated for 10 min at 90°C, loaded into the wells, and the gels run at 100 V. The gel was transferred onto a PVDF membrane at 220 mA overnight, and the membrane was then blocked with 5% dry milk (Carnation) and 1% BSA in TBS-T solution for 1 h. A primary antibody (1:200) was added to the solution and left overnight at 4°C (Li et al., 1995). The antibodies used were rabbit Anti-K Cadherin/CDH6 antibody (ab197845) (Abcam, Cambridge, CB2 0AX, United Kingdom), donkey anti-rabbit IgG-HRP: sc-2313 (Santa Cruz Biotech); Housekeeping: GAPDH mouse antibody (1:20,000 dilution), 2nd for housekeeping: GAPDH donkey anti-mouse: 1:1,500 dilution (Santa Cruz Biotechnology, Inc., Dallas, Texas 75,220, USA); Invitrogen OCT4 mouse Monoclonal Antibody (9B7) (1:1,000 dilution) (Thermo Fisher Scientific Inc., USA); and YAP (D8H1X) XP^®^ Rabbit mAb #14074 (Cell Signaling Technology, Inc., Danvers, MA 01923, USA). The membrane was washed three times with TBST, and a secondary HRP-conjugated antibody was then added (1:10000) and incubated for 2 h (Vu et al., 2015). The PVDF membrane was again washed three times with TBST. A chemiluminescence solution (Amersham) was added to the membrane, and the protein complexes were visualized using a chemiluminescent device (Li et al., 1998). The resulting blots were representative of three independent experiments.

### Clinical Data Analysis

Clinical data analysis was performed using the Oncomine database platform (Chen et al., 2018) and the NIH Genomic Data Commons portal and Cancer Genome Atlas (TCGA) to review previous cancer studies systematically. We surveyed *YAP1, CDH6,* and *OCT4* in all cohorts available from Oncomine and TCGA and identified 56 clinical studies in which at least one of those genes was significantly up-regulated in solid tumors. Fold change and *p*-values (*p* < 0.05) were evaluated based on differentially expressed genes (DEG) from comparisons of solid tumors *vs*. normal tissues.

## Results

### Osteogenesis Associated With MSC is a Stepwise Process

**FIGURE 1:**
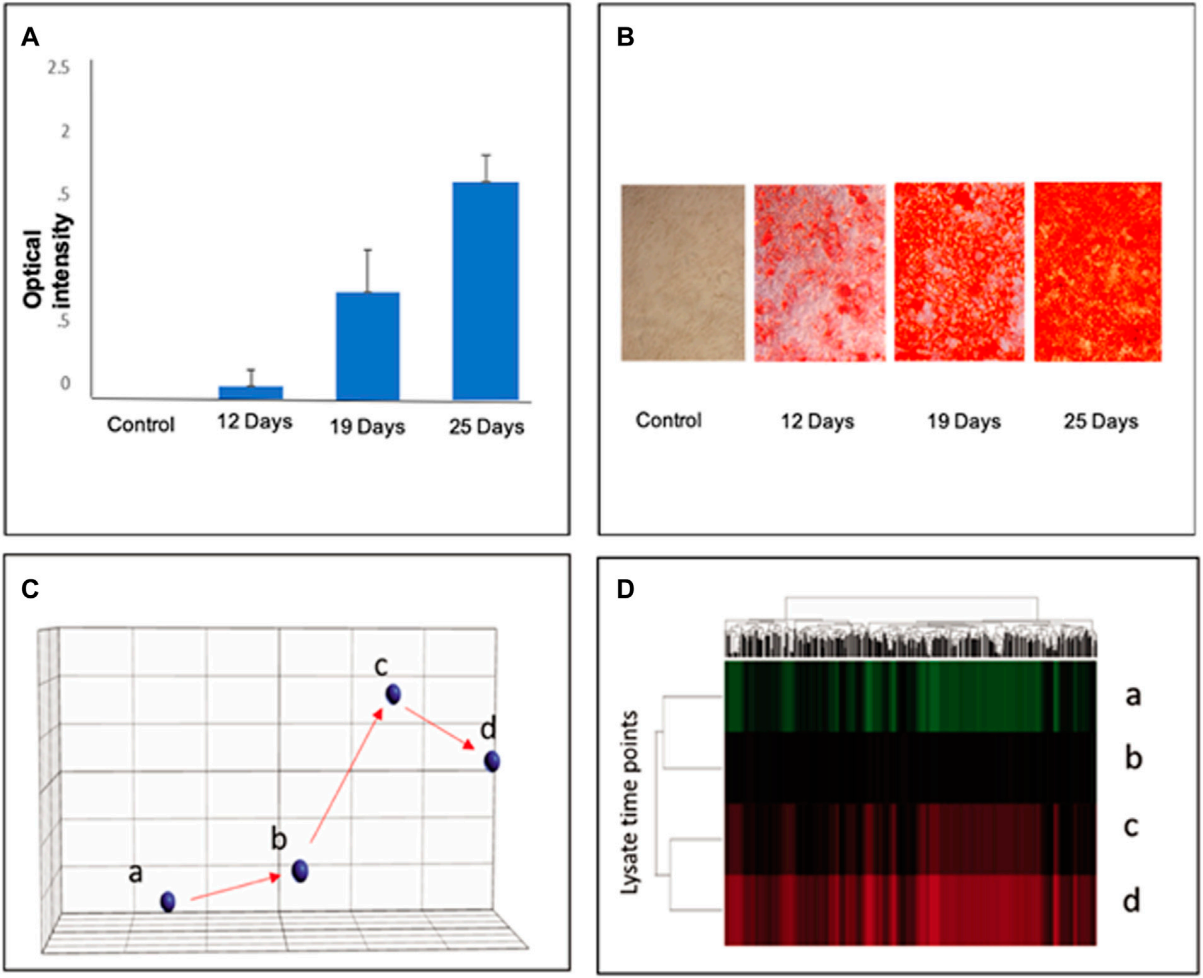
Stepwise differentiation of MSCs at days 12, 19, and 25. Alizarin Red staining confirmed the differentiation of MSCs into osteoblasts in culture to visualize calcium deposition. **(A)**: The staining intensity of Alizarin Red was quantified to reflect the percentage of osteogenic differentiation of MSC lysates; error bars represent s.e.m. **(B)**: Visual representation of differentiated cell density in culture. **(C)** Principal component analysis (PCA) of gene profiles from bone marrow MSC cell lysates at varying stages of differentiation: a–MSCs; b–D12; c–D19; D25 days after osteoinductive differentiation. **(D)** Hierarchical clustering shows significant differences among the three groups determined by ANOVA (*p* < 0.05) (Refer to Supplementary Table S1 for a total of 22,056 genes in the normalized counts and statistics).

Alizarin Red was used as a stage marker of bone matrix mineralization to quantify calcium deposition by colorimetric means. In MSC culture, 20% of cells had differentiated after 12 days, and 80% of cells were differentiated after 19 days. After 25 days, nearly 95% of cells were differentiated (Figures 1A,B) Aliquots from each group were harvested for bulk lysate RNA-seq, as well as for microfluidic single-cell capture and single-cell RNA-seq analysis, as described previously (Chen et al., 2018). Gene expression profiles from different time points indicated that the cells were temporally stepwise differentiated toward osteoblasts with distinct gene expression profiles from distinct stages (Figures 1C,D). Note that a total of 22,056 genes was used to analyze the normalized counts and statistics (Refer to Supplementary Table S1).

### Single-Cell Transcriptome Analyses Reveal Genetic Profiles of MSCs

**FIGURE 2:**
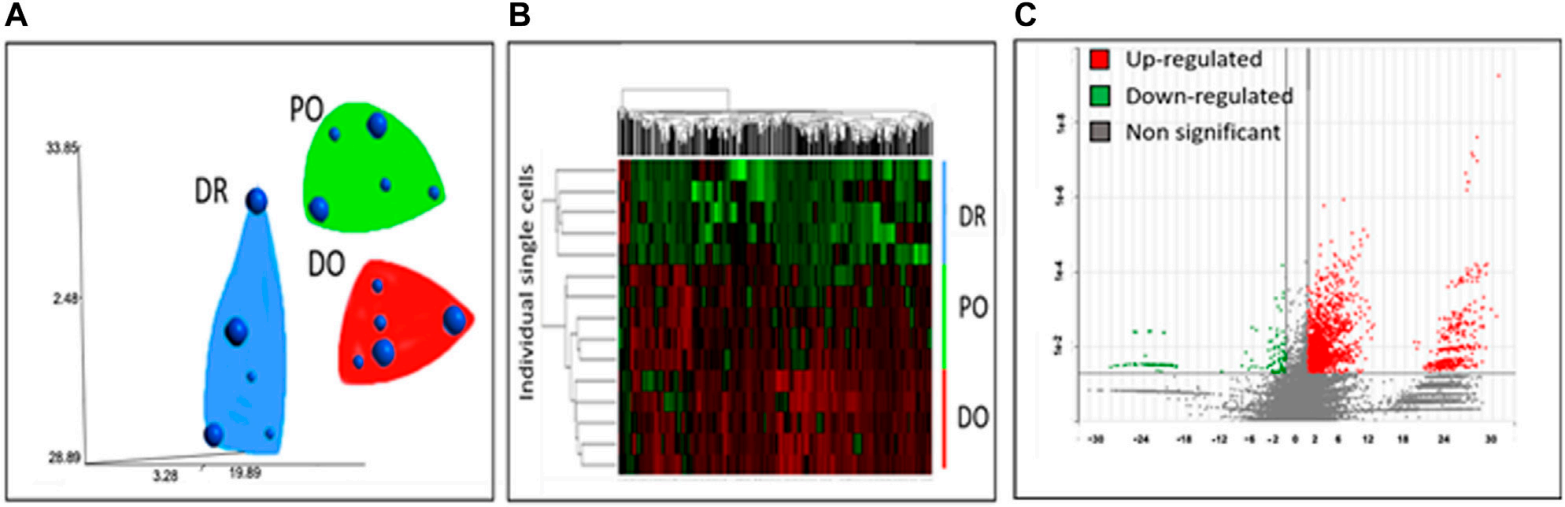
Clustering of differentiating single MSCs. Single-cell transcriptome analyses of gene expression profiles group cells into 3 clusters: differentiated osteoblasts (DO), differentiation-resistant MSCs (DR-MSCs), and precursor osteoblasts (PO). **(A)** 2 Dimensional representation of T-distribution stochastic neighbor embedding (tSNE) of gene expression profiles associated with single-cell transcriptomes. Small spheres represent cells harvested on day 12. In contrast, large spheres represent single cells harvested on day 19. Groups were determined based on gene expression similarity **(B)** The genes represented in the hierarchical clustering were significantly different among the three groups. (*p* < 0.05 and >2fold change); hierarchical clustering clearly identifies three groups with separate gene expression profiles **(C)** Genes differentially expressed (*p* < 0.05, fold difference >2) between the differentiation resistant group (DR-MSCs) and the differentiated osteoblasts (DO). *Y*-axis represents log-transformed *p*-values, while *X*-axis shows fold change between groups. There were 3,126 genes upregulated in DO (red) and 117 downregulated in DO (green). Genes that were not significantly differentially expressed between the two groups are grey (Refer to Supplementary Tables S1–S3).

**FIGURE 3:**
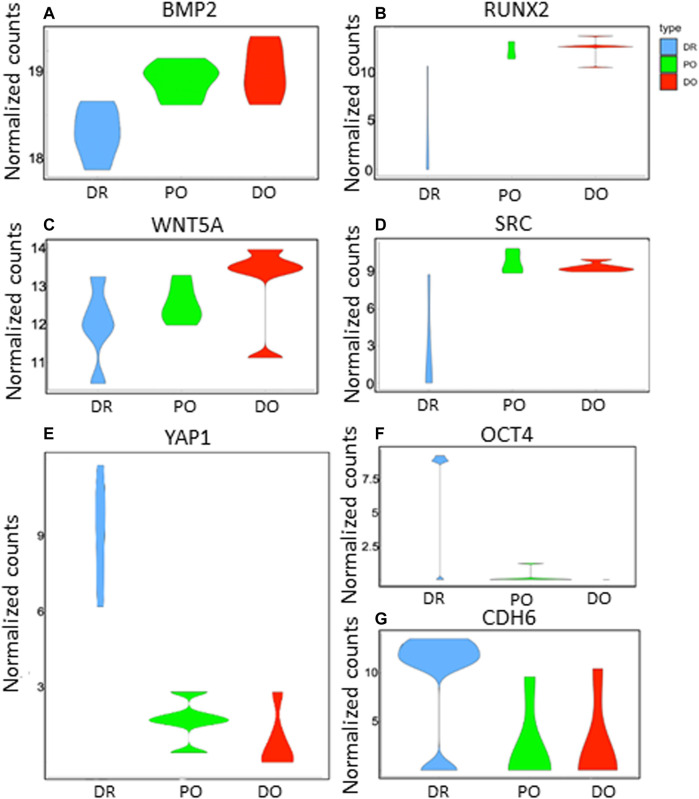
Gene expression profiles of DR, PO, and DO clusters. The violin plots show the distribution of key gene expressions in single cells from differentiation resistant MSCs (DR, blue), precursor osteoblasts (PO, green), and differentiated osteoblasts (DO, red). *p*-values represent differences of DR cluster compared to PO cluster and DO cluster respectively as follows: **(A)** BMP2 (3.8E-4; 2.9E-4), **(B)** RUNX2 (0.05; 0.04), **(C)** WNT5A. (0.04; 0.037), **(D)** SRC (4.8E-2; 3.2E-2). **(E)** YAP1 is significantly elevated in DR-MSCs compared to PO and DO (*p* = 0.03; *p* = 0.032, respectively). **(F)** Transcription factor OCT4 was significantly upregulated in DR-MSCs compared to PO and DO (*p* = 0.0168; *p* = 0.0161, respectively). **(G)** CDH6 expression was also markedly higher in the DR MSC cluster when compared to PO and DO (*p* = 0.0366; *p* = 0.0324, respectively).

As shown in Figure 2A-C, the differentiating MSCs were clustered by expression profile similarity into three clusters: 1) differentiated osteoblasts (DO), 2) differentiation/osteogenesis-resistant MSCs (DR- MSCs), and 3) precursor osteoblasts (PO). Comparing the 3 cell clusters revealed a dramatic up-regulation of osteoblast marker gene expression in the PO and DO groups but not in the DR MSC group. Compared to unmanipulated MSCs, a total of 152 down-regulated genes and 2,954 up-regulated genes were shown in the PO cluster (*p* < 0.05). Most interestingly, gene expression analysis identified 1780 genes with significantly different expression levels in the PO cluster than the DR-MSC cluster and identified 3,126 up-regulated and 117 down-regulated genes in the DO group compared to DR-MSCs (Figure 2C). To further characterize each of the three clusters, we profiled the relative expression of well-known mesenchymal and differentiated osteoblast markers such as Runt Related Transcription Factor (*RUNX2*), Bone morphogenic proteins (*BMPs*), and wingless (*WNT*)- related proteins (Grigoriadis et al., 1988) as well as previously characterized cytoskeletal proteins that are upregulated during the osteogenic differentiation process. Both the DO and PO groups showed a high expression of osteogenic transcription factor RUNX2, with no significant difference between the two groups from *p* = 0.77 to *p* = 0.75). However, *RUNX2* was differentially expressed between the DR and PO groups (*p* < 0.05) and between the DR and DO groups (*p* < 0.04). *BMP2, BMP4*, and *BMPR1B*, but not *BMP6*, were all differentially expressed in the PO and DO groups compared to the DR-MSC group (*p* < 0.01). Levels of BMP2 and BMP4 were significantly different between the PO and DO groups” from *p* = 0.3 to *p* = 0.006 and 0.04, respectively). *BMPR1B* was 3.4-fold higher in the DO group compared to the PO group (*p* < 0.05), suggesting a slight but significant difference in genetic profiles between the PO and DO groups (Figures 3A–D) (Refer to Supplementary Tables S1–S3). The single-cell transcriptome results of YAP1, CDH6, and Oct4 in differentiating MSCs indicated that YAP1 is significantly elevated in DR-MSCs compared to PO and DO (*p* = 0.03; *p* = 0.032, respectively) (Figure 3E). Transcription factor OCT4 was upregulated considerably in DR-MSCs compared to PO and DO (*p* = 0.0168; *p* = 0.0161, respectively) (Figure 3F). CDH6 expression was also markedly higher in the DR MSC cluster when compared to PO and DO (*p* = 0.0366; *p* = 0.0324, respectively) (Figure 3G).

### CDH6, YAP1, and OCT4 Participate in Solid Tumor Development

We evaluated the strong correlation of CDH6, YAP1, and OCT4 expression within solid tumors by analyzing 56 clinical cancer cohorts from Oncomine and TCGA databases. As shown in Table 1, YAP1, CHD6, and OCT4 were all highly co-expressed in 27 different cancers from 9 of the queried cohorts, including colorectal (*n* = 659), cecum (*n* = 237), renal (*n* = 11) pancreatic (*n* = 52), lung (*n* = 291), brain (*n* = 1711), bladder (*n* = 157), and cervical (*n* = 300) [24 [25 [26 [28 [30 [32 [33 [34 [35 (Skrzypczak et al., 2010). Based on these data, we identified that YAP1 and CDH6 were highly co-expressed in a total of 44 queried cohorts, while YAP1 and OCT4 were highly co-expressed in 32, and CDH6 and OCT4 in 31. In particular, we identified that YAP1 expression significantly changed in two large liver cancer cohorts (*p* = 4.4E-19 and *p* = 0.030, respectively) [ (Roessler et al., 2010) (Wurmbach et al., 2007). At the same time, CDH6 and OCT4 were both up-regulated significantly in liver cancer compared to normal controls. Moreover, YAP1, CDH6 and OCT4 were significantly up-regulated in cervical cancer (*p* = 0.038, 8.98E-7 and 0.002, respectively) (Scotto et al., 2008). Next, we wanted to test if this signaling pathway regulates the cellular functions with a well-characterized HeLa cell system established in our laboratory.

**TABLE 1:** 56 clinical cancer cohorts from Oncomine and TCGA databases.

Cancer type	*p*-value			Fold change			Patients	Ref
	YAP1	OCT4	CDH6	YAP1	OCT4	CDH6		
Cecum Adenocarcinoma *vs*. Normal	7.87E-09	2.13E-04	4.49E-06	1.834	1.759	1.52	237	NCI, (2015)
Cervical Squamous Cell Carcinoma *vs*. Normal	0.037	0.002	8.98E-07	1.091	1.061	1.1	100	Scotto et al. (2008)
Clear Cell Renal Cell Carcinoma *vs*. Normal	0.034	0.01	0.012	1.503	1.265	1.459	18	Lenburg et al. (2003)
Colon Adenocarcinoma *vs*. Normal	7.57E-15	1.45E-10	7.53E-07	1.79	2.24	1.486	237	NCI, (2015)
Colorectal Carcinoma *vs*. Normal	1.39E-07	0.001	1.14E-05	1.526	1.13	1.189	105	Skrzypczak et al. (2010)
Lung Adenocarcinoma *vs*. Normal	0.018	0.008	1.17E-15	1.028	1.029	1.2	291	Weiss et al. (2010)
Pancreatic Carcinoma *vs.* Normal	2.99E-05	0.038	0.031	1.958	1.194	1.47	52	Pei et al. (2009)
Rectal Adenocarcinoma *vs.* Normal	2.38E-22	3.17E-22	1.30E-16	1.799	2.514	1.51	130	Gaedcke et al. (2010)
Rectosigmoid Adenocarcinoma *vs.* Normal	5.25E-06	2.55E-06	1.48E-04	2.132	1.591	1.412	105	Kaiser et al. (2007)
Superficial Bladder Cancer *vs.* Normal	0.019	1.08E-11	0.042	1.228	4.969	1.543	157	Sanchez-Carbayo et al. (2006)
Teratoma, NOS *vs*. Normal	7.73E-09	0.024	1.06E-04	2.771	1.159	1.329	107	Korkola et al. (2006)
Gastric *vs.* Normal	0.009	4.70E02	0.011	1.024	1.037	1.030	291	Deng et al. (2012)
Glioblastoma *vs*. Normal	3.08E-14	7.92E-05	2.83E-03	2.265	1.653	1.452	180	Sun et al. (2012)

### CDH6/YAP1/OCT4 Interaction in Solid Tumor Cell Lines

**FIGURE 4:**
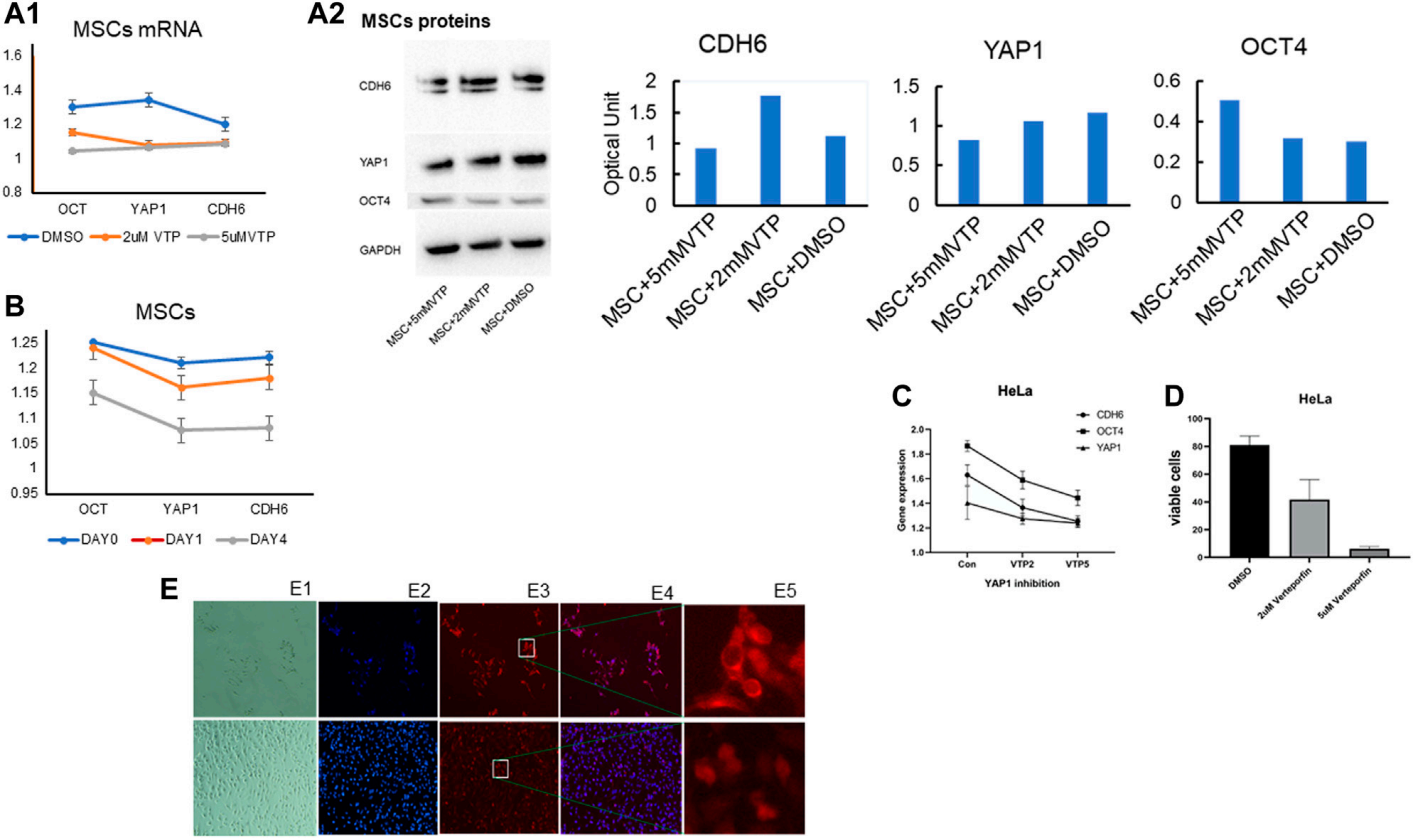
**V**erteporfin significantly decreases solid tumor cell viability and inhibits YAP1. **(A)** MSCs were harvested on Day 12 following incubation in a differentiation medium. CDH6, YAP1, and OCT4 transcripts were all significantly down-regulated by 2 M Verteporfin in a time-dependent manner **(A1)** and the corresponding proteins by Western blotting (Blots are representative of three independent experiments, adjacent to the quantification of the related proteins) **(A2)**. Verteporfin significantly downregulated **(B)** Transcripts for OCT4 in a dose-dependent manner (at both 2 and 5 M doses), but no significant changes were observed for YAP1 or CDH6 [Note that all gene expression levels are indicated expression levels relative to the untreated controls]. **(C)** Transcripts for CDH6 and OCT4 by PCR were significantly decreased in tumor cells by 2 M verteporfin compared to controls, but no significant differences were observed for YAP1. [All the above qPCR results were normalized against beta actin]. **(D)** HeLa cells were incubated in 2 and 5 M concentrations of verteporfin for 24 h and harvested. The trypan blue dye exclusion test was used to assess the viability of cells cultured with and without verteporfin. Cells were counted under a hemocytometer. Cell viability in both cancer cell lines was inversely correlated to increasing doses of verteporfin. Cell viability was significantly decreased (*p* < 0.05) with 2 and 5 M concentrations of verteporfin when compared to controls across both cell lines. **(E)** Effects of YAP1 inhibitor verteporfin on HeLa cells after 24 h incubation. Top panels E1 to E5: **(E1)** Bright-field image; **(E2)** NucBlue staining for live cells; **(E3)** YAP1 fluorescent staining; **(E4)** merged NucBlue (blue) and YAP1 antibody staining (red). **(E5)** Images with higher magnification show the location of YAP1. Lower panels of the E1 to E5 are the corresponding controls showing cells cultured with DMSO. Verteporfin administered at 2uM in cell culture substantially reduced the number of viable cells compared to DMSO controls. Verteporfin significantly reduced YAP nuclear localization, and cells cultured with DMSO showed YAP1 staining mostly localizing to the nucleus and staining overlapped with NucBlue.

We previously found that *CDH6, YAP1,* and *OCT4* transcripts were highly expressed in the HeLa (cervical) cancer cell line (Chen et al., 2018). Here, we found that transcript levels for *CDH6* and *OCT4* were all significantly (*p* < 0.05) reduced in MSCs (Figures 4A1,B) in the presence of 2 mM or 5 mM verteporfin. However, only YAP1 appeared downregulated at the protein levels (Figure 4A2). Similar patterns of transcript levels were observed in HeLa cells (Figures 4C,D) by the addition of 2 μM or 5 μM verteporfin in the culture in a time-dependent manner and a dose-dependent manner (Figure 4B). Additionally, following 24 h exposure to Verteporfin at a dosage of 2 mM or 5μM, cell viability was significantly decreased (*p* < 0.05) compared to controls (Figure 4D). Immunofluorescence staining showed that YAP1 was mainly localized to nuclei in the control cultures. The staining for YAP1 was overlapped with NucBlue, whereas YAP1 in verteporfin-treated cells was localized primarily for cytosolic regions (Figures 4E1–E5). The addition of Verteporfin to the cell culture at 2 or 5 μM gradually inhibited YAP1 nuclear localization and reduced the transcription of *CDH6* and *OCT4* (Figures 4E1–E5). We noticed that Verteporfin is known to reduce YAP1 *via* induction of the SUMOylation of YAP1 (Wang et al., 2020), suggesting a possible way to regulate the pathophysiology.

### Pathway Analyses of Differentiating MSCs

**FIGURE 5:**
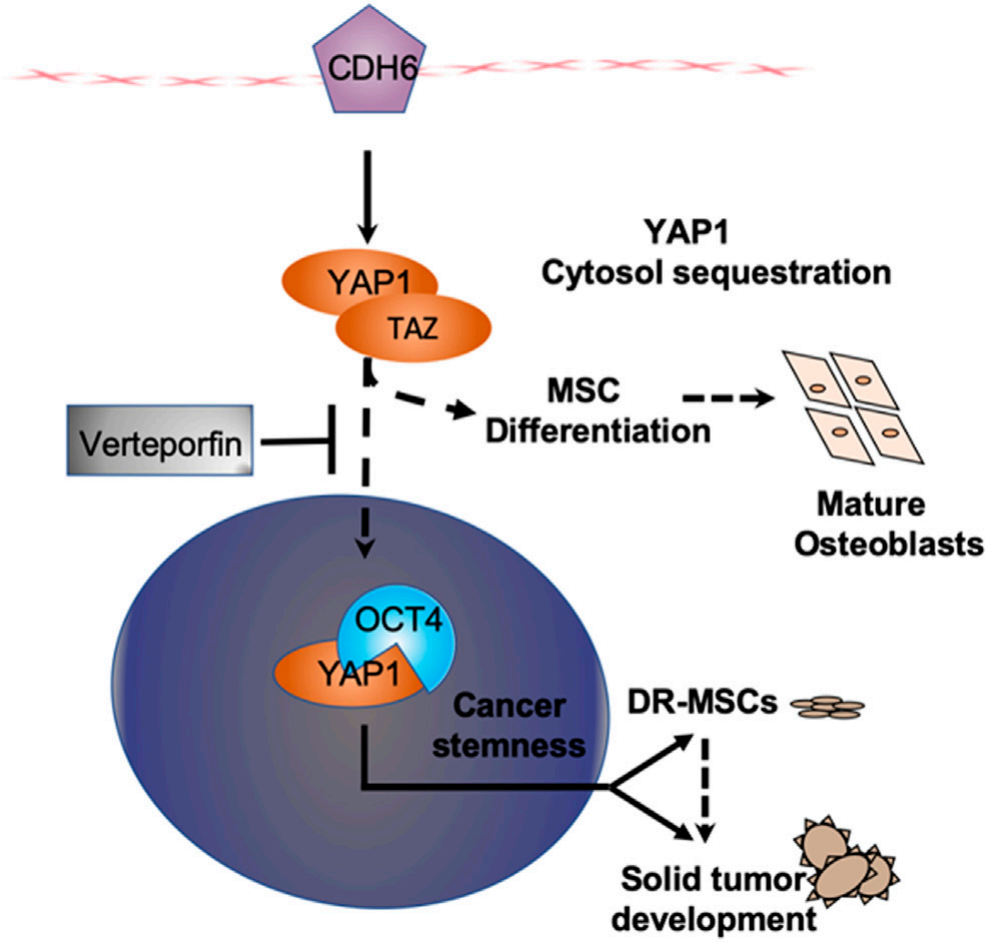
YAP1-related signaling and associated proteins CDH6 and OCT4. YAP1-related signaling and associated proteins CDH6 and OCT4 were significantly up-regulated (*p* = 4.25E-8). Upon mechanical strain or cellular contact, cadherin can induce YAP1/TAZ nuclear localization, upregulate transcription of OCT4, and promote MSC stemness. Verteporfin reduces levels of YAP1, CDH6, and OCT4, inhibits YAP1 nuclear localization, induces YAP1 cytosolic sequestration, and promotes MSC differentiation.

Using Ingenuity Pathway Analysis (IPA^®^), we identified the most prominent molecular signaling mechanisms involved in the differentiation of MSCs into mature osteoblasts. We identified pathways related to canonical PI3K signaling (*p* = 1.65E-06), PKA (*p* = 2.68E-06), AKT (*p* = 3.72E-29), Estrogen (4.8E-06), and ERK/MAPK (*p* = 1.87E-05) as being significantly involved in the differentiation of MSCs into mature osteoblasts. In particular, YAP1 signaling (*p* = 4.25E-8) (Figure 5) was significantly up-regulated in differentiation-resistant MSCs when compared to PO and DO groups. The most significant difference between PO and DO cohorts was eIF2 signaling with two-fold up-regulation (*p* = 1.6E-16) in DO compared to PO.

## Discussion

YAP1 is well known to promote cancer formation, tumor progression, and metastasis [41 (Stanger, 2012) but is less known to play a role in MSC-involved tumorigenicity. It has been reported that MSC osteogenesis is regulated by the FAK/RhoA/YAP1 pathway (Chang et al., 2018). Our study identified a subpopulation (i.e., subclone) of MSCs, which may contribute to MSC--involved tumorigenicity *via* YAP1 signaling. We used single-cell transcriptomes to identify heterogeneity within an MSC subpopulation and found DR-MSCs with high YAP1 expression and resistance to osteogenesis, cells that are blocked at specific differentiation stages during osteogenesis. Those DR-MSCs are similar to leukemia cells blocked during HSC differentiation [ (Chopra and Bohlander, 2019) (Nowak et al., 2009). We analyzed this subpopulation and found the up-regulation of YAP1, CDH6, and OCT4 in those DR MSCs. CDH6 is an EMT biomarker often highly expressed in solid tumors and enhances cancer invasiveness and metastasis [ (Casal and Bartolomé, 2019) (Sancisi et al., 2013). It has been reported that YAP1 and cadherins collaboratively affect cancer mechanotransduction (Ma et al., 2018). Cell adhesion-initiated mechanical strain induces cadherin-dependent YAP1 activation to drive cell cycle entry; in this way, activated YAP1 may represent a master regulator of cancer-driven mechanical strain-induced cell proliferation.

On the other hand, cadherins provide signaling centers required for cellular responses to the externally applied force (Benham-Pyle et al., 2015). Thus, a network interaction between YAP1 and CDH6 signaling may be involved in G protein-coupled receptor-mediated kinase cascade elements, regulated by intrinsic and extrinsic signals, such as mechanical force and cell-cell contact polarity, energy status, stress, and many diffusible hormonal factors. OCT4 is well known for being a stem cell marker and plays a crucial role in cancer progression (Kim and Nam, 2011) and drug resistance [ (Cordenonsi et al., 2011) (Tang et al., 2015). After 12 days in culture, only about 20% of MSCs were differentiated in our study, but this percentage increased to 80% by 19 days. YAP1, CDH6, and OCT4 expression reduced gradually during stepwise differentiation, implying CDH6/YAP1/OCT4 signaling interactions are relevant to MSC differentiation, possibly revealing negative regulation. Differences between unmanipulated MSCs and DR-MSCs were significant for CDH6 (*p* = 0.0441) but not for OCT4 (*p* = 0.0724), suggesting that DR MSCs have similar stemness to unmanipulated MSCs. YAP1 inhibition by small-molecule inactivation of YAP1 or associated proteins has become an increasingly promising therapeutic strategy to treat aggressive cancers (Tremblay et al., 2014). Specific reagents, including dasatinib, pazopanib, and Verteporfin, inhibit the nuclear localization of YAP1 in the nanomolar to the micromolar range and interfere with the migration of various cell lines from solid tumors (McCaig et al., 2012). Our study inhibited YAP1 activity with Verteporfin, inducing sequestration of YAP1 to the cytoplasm (Wang et al., 2016) in MSCs, and solid tumor cell lines. YAP1 inhibition by Verteporfin impairs TGF-β-induced Smad2/3 nuclear accumulation and transcriptional activity to attenuate renal fibrosis (Szeto et al., 2016) in response to mechanoregulators of organ stiffening.

Furthermore, transcription of *YAP1, OCT4,* and *CDH6* was reduced time-dependent. Interestingly, increasing Verteporfin’s dose dramatically affected OCT4 expression on Day 4 but had no significant impact on YAP1 or CDH6. Since verteporfin inhibition of YAP1 is induced by YAP1 cytosol sequestration, increased doses of Verteporfin might not significantly affect *YAP1* transcription. Moreover, CDH6 is a membrane receptor located upstream of YAP1 signaling, so its changes might not be immediately reflected by *YAP1* transcriptional activity. To our knowledge, a few studies mentioned human MSC-involved tumorigenicity [ (Røsland et al., 2009) (Rubio et al., 2005). However, the molecular mechanism of MSC-involved tumorigenicity remains elusive. Our study suggests that YAP1 overexpression could block MSC differentiation and lead to tumorigenesis with a mechanism similar to leukemia formation due to blockage of HSC differentiation [ (Chopra and Bohlander, 2019) (Nowak et al., 2009).

YAP1 signaling has been reported to be highly active in solid tumors [ (Warren et al., 2018) (Stanger, 2012). Based on our MSC data, tumorigenesis driven by CDH6/YAP1/OCT4 interactions in DR MSCs may be similar to leukemia, causing blockage of cell differentiation in various stages of hematological development [ (Lee et al., 2019) [ (Chopra and Bohlander, 2019) (Li et al., 2013). Therefore, we confirmed the co-expression of YAP1, CDH6, and OCT4 in 56 clinical cohorts, including various solid tumors (Table.1). Among the representative solid tumors, YAP1, CDH6, and OCT4 were significantly up-regulated, implying that interactions among them in a subpopulation of MSCs could be responsible for the genesis of solid tumors (Figure 5). Given that these clinical data were obtained from bulk lysates with cancer and normal cells mixed, such statistically significant findings suggest that even slight up-regulation of transcription could reflect dramatic changes in the cancer stem cell subpopulation. To further investigate the role of CDH6/YAP1/OCT4 signaling in solid tumors, we performed *in vitro* experiments using a HeLa solid tumor cell line. We rationalized that a cervical cancer cell HeLa with a different origin than MSC could validate the YAP/FOXM1 axis’s functional role based on our previous studies showing the molecular similarity across the organ-based cancer classifications (Li et al., 2018).

Interestingly, we found that YAP1, CDH6, and OCT4 were highly expressed in HeLa cells. Following verteporfin treatment, YAP1 expression was still detected in the cytoplasm 24 h later, but its transcription ability was lost as it could not transfer to the nucleus. Only a fraction of cancer cells highly expressed YAP1, implying that only a subpopulation possesses cancer stem cell (CSC)-like characteristics similar to DR MSCs. YAP1 and OCT4 displayed co-expression in solid tumors, suggesting YAP1 is likely a CSC biomarker as well. If YAP1 is lost, CSC self-renewal potential will decrease (Bora-Singhal et al., 2015). It has been reported that YAP1 inhibition by Verteporfin does not enhance the antitumor efficacy of temozolomide in glioblastoma (Liu et al., 2020). However, our study found that YAP1 inhibition by verteporfin administration reduced cancer cell viability and stemness and reduced levels of CDH6 and OCT4. Therefore, even though the evidence for the relationship between CDH6/YAP1/OCT4 expression is inconclusive, our results indicate that YAP1 expression significantly correlates with CDH6/OCT4 expression and possibly affects the development of solid tumors through affecting cell proliferation, survival, mobility, and stemness. Further studies may help develop technology to eliminate those MSC-involved tumorigenic subclones (Li et al., 2012) for therapeutics in improving the efficacy of subclonal targets (Li et al., 2018).

## Conclusion

Our study illustrated gene expression profiling of MSCs through the stepwise (i.e., temporal) process of differentiation to the osteogenic lineage. All CDH6/YAP1/OCT4 molecules were highly expressed in DR-MSCs, but not differentiated PO or DO. YAP1 inhibitor promoted MSC differentiation and reduced transcription of YAP1, CDH6, and OCT4 in a time- and dose-dependent manner. Interestingly, only a fraction of solid tumor cells displayed high *YAP1* gene expression and evidence of cancer stemness similar to the profiles of DR-MSCs. It remains unclear how YAP1 nuclear trans-localization affects CH6 and OCT4; however, taken together, the results of previous reports [ (Røsland et al., 2009) (Rubio et al., 2005) and our current study suggest that the detrimental effects of MSCs may be due to a subpopulation (i.e., a subclone) of DR-MSCs that potentially contribute to the development of solid tumors due to blockage of differentiation similar to leukemia formation [ (Chopra and Bohlander, 2019) (Nowak et al., 2009). To our knowledge, this is the first report that CDH6/YAP1/OCT4 transcriptional expression can play an essential role in both DR MSCs and solid tumors. Further investigation is needed to clarify how these molecules interact in MSC--involved tumorigenesis by blocking MSC differentiation.

## Data Availability

The original contributions presented in the study are included in the article/Supplementary Material, further inquiries can be directed to the corresponding authors.

## Abbreviations

CSC, cancer stem cells; DO, differentiated osteoblasts; DR, osteogenesis-resistant MSCs; MSC, mesenchymal stem cells; PO, precursor osteoblasts.
